# Pollination services enhanced with urbanization despite increasing pollinator parasitism

**DOI:** 10.1098/rspb.2016.0561

**Published:** 2016-06-29

**Authors:** Panagiotis Theodorou, Rita Radzevičiūtė, Josef Settele, Oliver Schweiger, Tomás E. Murray, Robert J. Paxton

**Affiliations:** 1 General Zoology, Institute for Biology, Martin Luther University Halle-Wittenberg, Hoher Weg 8, 06120 Halle (Saale), Germany; 2 German Centre for Integrative Biodiversity Research (iDiv) Halle-Jena-Leipzig, Deutscher Platz 5e, 04103 Leipzig, Germany; 3 Department of Community Ecology, Helmholtz Centre for Environmental Research-UFZ Halle, Theodor-Lieser-Strasse 4, 06120 Halle, Germany; 4 National Biodiversity Data Centre, Beechfield House, WIT West Campus, Waterford, Co., Waterford, Ireland; 5 Molecular Evolution and Animal Systematics, Institute of Biology, University of Leipzig, Talstrasse 33, 04103 Leipzig, Germany; 6 ESCALATE, Department of Computational Landscape Ecology, Helmholtz Centre for Environmental Research-UFZ Leipzig, Permoserstrasse 15, 04318 Leipzig, Germany; 7 Department of Zoology, Faculty of Natural Sciences, Vilnius University, M.K. Čiurlionio 21/27, 03101 Vilnius, Lithuania

**Keywords:** *Bombus*, *Crithidia bombi*, *Nosema bombi*, local habitat, land-use change, plant–animal interactions

## Abstract

Animal-mediated pollination is required for the reproduction of the majority of angiosperms, and pollinators are therefore essential for ecosystem functioning and the economy. Two major threats to insect pollinators are anthropogenic land-use change and the spread of pathogens, whose effects may interact to impact pollination. Here, we investigated the relative effects on the ecosystem service of pollination of (i) land-use change brought on by agriculture and urbanization as well as (ii) the prevalence of pollinator parasites, using experimental insect pollinator-dependent plant species in natural pollinator communities. We found that pollinator habitat (i.e. availability of nesting resources for ground-nesting bees and local flower richness) was strongly related to flower visitation rates at the local scale and indirectly influenced plant pollination success. At the landscape scale, pollination was positively related to urbanization, both directly and indirectly via elevated visitation rates. Bumblebees were the most abundant pollinator group visiting experimental flowers. Prevalence of trypanosomatids, such as the common bumblebee parasite *Crithidia bombi,* was higher in urban compared with agricultural areas, a relationship which was mediated through higher *Bombus* abundance. Yet, we did not find any top-down, negative effects of bumblebee parasitism on pollination. We conclude that urban areas can be places of high transmission of both pollen and pathogens.

## 1. Introduction

Loss of biodiversity in general, and of pollinator diversity in particular, is thought to be primarily driven by human-induced land-use change [1,2]. (Semi-) natural habitats, rich in diverse floral food resources and pollinator nesting opportunities, are fragmented and degraded during conversion to highly impervious urban and intensively managed agricultural areas [3]. Thus, impacts of both increasing urbanization and agricultural intensification on wild pollinator communities are considered to be, on the whole, negative [4–6].

However, responses may vary depending on the magnitude of the land-use change and type of land-use conversion [7], leading to neutral or even positive effects on pollinators [8–10] and the ecosystem service of pollination [11,12]. Moderate human land-use can potentially support pollinator populations at the landscape scale by increasing heterogeneity of the surrounding habitat mosaic and thus enhance availability and accessibility of suitable habitats [2]. At the local scale, moderate human land-use can also provide alternative foraging and nesting resources for bee pollinators [2,9,13]. Indeed, increasing evidence suggests that moderately urbanized environments can facilitate pollinator persistence. For example, flower-rich suburban gardens have been found to promote quicker bumblebee colony growth [14] and higher nest densities compared with farmland [15] or other types of rural habitats [16]. Appropriately managed urban areas are therefore potentially important reservoirs of pollinator biodiversity [17,18].

In addition to land-use change, pathogens can also negatively affect pollinator populations and pollination. Pathogens are known to reduce pollinator fitness and abundance and alter their foraging behaviour [19], thus potentially causing cascading, negative multi-trophic effects on pollination. For example, Gillespie & Adler [20] showed that the seed set of plants (e.g. *Trifolium pratense*) associated with bumblebee (*Bombus* spp.) visitation was negatively correlated with prevalence of the native microsporidian parasite *Nosema bombi* in these pollinators.

However, no study has so far considered top-down effects of parasitism on pollination across changing landscapes and thus the interactive effects of anthropogenic land-use change and parasitism on mutualistic plant–pollinator interactions and pollination service provision. For example, as anthropogenic land-use can influence pollinator abundance, it could indirectly affect the prevalence of pathogens, which, all else equal, would be predicted to be higher in high density host populations and vice versa [21]. As urbanized areas seem to support high densities of some pollinators such as bumblebees [16,22], this could potentially further promote parasite abundance and transmission [21] and reduce pollination service provision.

Bumblebees offer us an excellent model system to study the indirect effects of parasitism on mutualisms [20]. The majority of *Bombus* spp., are generalist pollinators, foraging on many native wild and crop plants in diverse terrestrial habitats in temperate regions [15]. Furthermore, some species are quite resilient to land-use change and can be found across a gradient of habitat disturbance. Bumblebees are also attacked by a number of parasites, including the trypanosomatid *Crithidia bombi* and the microsporidium *Nosema bombi*. *Crithidia bombi* is a common gut parasite that affects colony reproduction and foraging performance [19,23–25]. The less common *N. bombi* infects the entire animal and has been shown to reduce worker survival and colony fitness [26–28]. Thus, these two parasites could potentially influence both the quantity and quality of plant–pollinator interactions and alter the provision of pollination service to plants [20].

We conducted an empirical study on flower visitation and pollinator parasitism across an array of sites varying in human land-use from agricultural to urban and, at the same sites, evaluated pollination service provision using experimental arrays of wild flowers. We then used piecewise structural equation modelling (SEM) (i) to examine how an agricultural to urban land-use gradient and local habitat features affected pollinator visitation rates and parasitism of bumblebees and (ii) to determine the interacting impacts of bumblebee abundance and parasitism on the ecosystem service of pollination of those experimental plant communities.

## 2. Methods

### (a) Study system and sites

**Figure 1.**
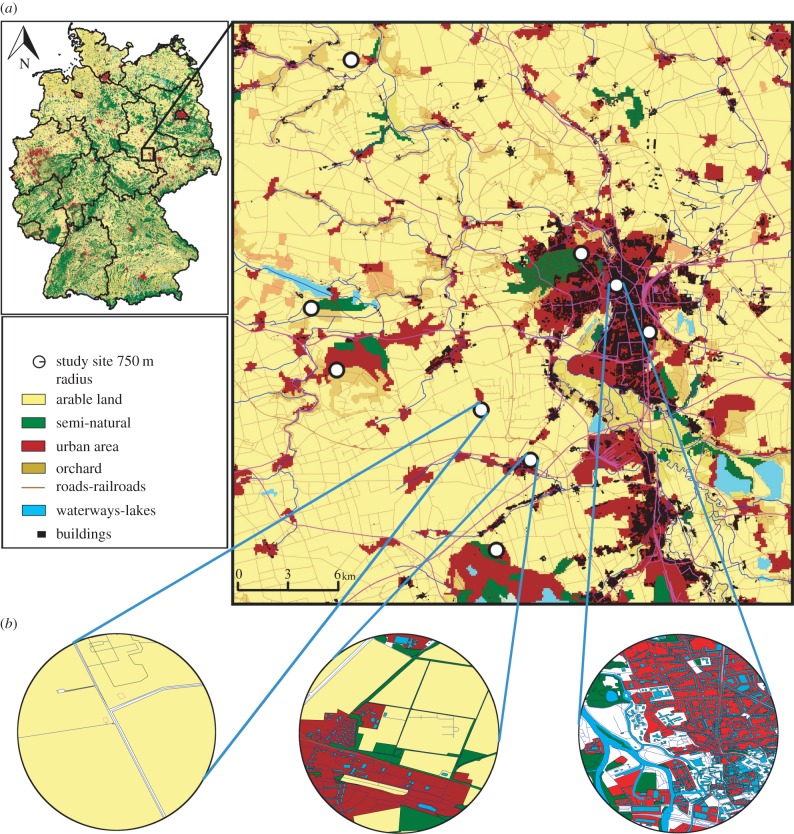
(*a*) Study area and study sites in the federal state of Saxony-Anhalt (Germany) in the surroundings of Halle (Saale); (*b*) examples of three study sites, showing their landscape heterogeneity and composition within a 750 m radius of a site's geographical centre.

Fieldwork was conducted in July and August 2013 at nine independent sites within the federal state of Saxony-Anhalt, Germany. Sites were selected using land cover maps within a Geographic Information System (GIS), to differ in their degree of anthropogenic land-use. Within each site, we selected a 25 × 25 m plot near its geographical centre with diverse floral resources, and ensured a minimum distance of 3 km between sites (figure 1 and see electronic supplementary material, table S1). Although other types of habitat were present, urban and agricultural land-use constituted more than 75% of the landscape surrounding all sites.

We used greenhouse-raised *Trifolium pratense*, *Trifolium repens*, *Borago officinalis*, and *Sinapis alba* plants as phytometers at all nine sites and evaluated their pollination success to estimate the provision of pollination services. All plant species are insect-pollinated and self-incompatible [29,30], differing in their dependence on pollinators with short (for the plants *B. officinalis* and *S. alba*) and long (for *Trifolium* spp.) mouthparts. Seeds of all plants were obtained by a local seed provider (Rieger Hofmann GmbH). After germination, seedlings were grown for two months in an insect-free greenhouse before placement at study sites.

Five potted plants per species with already open flowers marked with coloured tape were placed out at each field site for four days, facing south to reduce the effects of differential shading (*B. officinalis* and *S. alba* in July, *Trifolium* spp. in August). Plants were randomly placed at 1 m distance along a transect of 10 × 1 m within each plot. We monitored all flying insects visiting experimental plants for a single day at each site. We visited our sites in a random order between 6 July 2013 and 19 August 2013. We needed a total of 32 days to visit all sites across our gradient. Individual plants were observed for 20 min twice per site, once in the morning and once in the afternoon, for a total of 200 min observation time per focal plant species per site (for sampling effort-based flower visitor morphogroup accumulation curves, see electronic supplementary material, figure S1), between 09.00 and 17.00 h, on dry, warm, non-windy days (electronic supplementary material, table S1). Visitor identity (morphogroup: Coleopteran; hoverfly; other Dipteran; Lepidopteran; bees of the families Andrenidae, Colletidae, or Halictidae; *Bombus* spp.; honeybee) and duration of visit, defined as the total time the visitor contacted reproductive structures of the flower, were also recorded. From these data, we calculated the visitation rates of all flower visitors for each experimental plant species, as the number of visitors per flower unit per minute. Furthermore, we calculated the visitation rates of *Bombus* spp. and used them as a surrogate for *Bombus* abundance at each site.

Once field experiments were finished, focal plants were returned to the insect-free greenhouse until seeds were formed. Seeds from marked flowers were counted at the level of the floral display unit and used as a measure of the ecosystem service of pollination. An additional greenhouse experiment was performed to test the pollinator dependency of all experimental plant species. Five plants of each species were maintained during the entire flowering period in the insect-free greenhouse. Flowers were marked, and seed set was assessed in the same way as in field experimental plants.

### (b) Local and landscape variables

At each site, we quantified local flowering plant species richness, as an estimator of floral resource availability, and percentage of bare soil, as an estimator of nesting resource availability for ground-nesting bees (Andrenidae, Colletidae, Halictidae), using 10 1 m^2^ quadrats randomly placed in each 25 × 25 m plot. At each sampling site, we also quantified habitat composition at five spatial scales at radii of 500, 750, 1 000, 1 500, and 2 000 m. For each sampling site, we quantified habitat composition at all scales using Quantum GIS [31] and land-cover data obtained from Geofabrik GmbH (http://www.geofabrik.de/); electronic supplementary material, table S2). Landscape diversity (*H_s_*) was calculated using the Shannon index: *H_s_* = −∑*p_i_ ×* ln *p_i_*, where *p_i_* is the proportion of each land-cover type *i* [32]. To identify the most appropriate scale for landscape analysis, we correlated the pollinator visitation rates with landscape diversity at each of our study sites at the five scales and compared the resulting correlation coefficients. Correlation coefficients peaked at 750 m (electronic supplementary material, table S3), which was then chosen as the spatial scale for subsequent landscape-scale analyses.

To further examine the effects of anthropogenic land-use, we calculated a land-use index, ranging from pure agricultural (−1) to pure urban (+1), based on the proportional area of each land class within a 750 m radius (figure 1 and electronic supplementary material, table S1).

### (c) Bumblebee parasitism

Both female and male bumblebees of the morphogroups *Bombus terrestris*/*lucorum, Bombus pascuorum*, and *Bombus lapidarius/soroeensis proteus* were collected from each site between 1 and 19 August 2013. Sampling was not quantitative; rather we aimed to collect reasonable sample sizes of both sexes of each *Bombus* morphogroup so as to determine pathogen prevalence across males and females of each morphogroup. At each site, bees were sampled within a 500 m radius from established experimental plants. We sampled female and male individuals. Bees were kept in 100% ethanol and stored at −20°C. In total, we sampled and analysed 164 females and 150 males from all sites and *Bombus* morphogroups (electronic supplementary material, table S4).

We took pictures of each bumblebee's forewings using a digital camera mounted on a dissection microscope (Olympus DP21). Each individual was scored based on a four-point wing wear scale, and the score obtained was used as a surrogate for age in subsequent statistical analysis [33].

To determine the presence of *Crithidia* and *Nosema*, DNA of hosts and parasites was extracted using a modified Chelex protocol [34]. We homogenized the abdomen of each bee in 500 µl sterile distilled water, and 200 µl of each homogenate was centrifuged for 10 min at 20 000 *g*. The supernatant was then discarded and the remaining pellet was further homogenized in 100 µl of a 5% Chelex solution and 5 µl of 1% proteinase K. Samples were processed in a thermocycler using the following programme: 1 h at 55°C; 15 min at 99°C; 1 min at 37°C; and a final step for 15 min at 99°C. Chelex beads were then removed from the DNA samples by centrifugation at 12 000 *g* for 5 min. DNA was stored at –20°C until further processing. Polymerase chain reactions (PCRs) were conducted using primers that target portions of the small subunit rRNA region of each parasite taxon. For *N. bombi* detection, we used the primers NbombiSSUJf1 (CCATGCATGTTTTTGAAGATTATTAT) and NbombiSSUJr1 (CATATATTTTTAAAATATGAAACAATAA) [35] and for *Crithidia* spp. detection (including *C. bombi*), we used the primers SEF (CTTTTGGTCGGTGGAGTGAT) and SER (GGACGTAATCGGCACAGTTT) [36]. We performed separate reactions for each parasite taxon in a Biometra TProfessional thermocycler using 2 µl DNA template, 2X Promega PCR buffer, 0.4 µM of each primer, 0.2 mM of each dNTP (deoxynucleotide), and 0.5 U of GoTaq Polymerase (Promega) to a total volume of 10 µl. The following thermal cycling programme was used for the PCRs: initial denaturation step of 94°C for 2 min, followed by 40 cycles of 94°C for 45 s, annealing temperature at 50°C (NbombiSSUJf1/NbombiSSUJr1) or 57°C (SEF/SER) for 45 s and 72°C for 45 s, plus a final extension step of 72°C for 5 min. PCR amplicons were visualized on a QIAxcel (Qiagen) capillary fragment analyser, using an acceptance threshold of 0.1 relative fluorescence units.

### (d) Statistical analysis

Prior to statistical analysis, we used Mantel tests and spline correlograms (R package ‘ade4’, [37]; package ‘ncf’, [38]) to test for potential spatial autocorrelation in our dataset. There was no significant spatial autocorrelation for seed set, visitation rates, and prevalence of each *Bombus* spp. parasite (*p* > 0.05; electronic supplementary material, table S5).

To reduce any effect of multi-colinearity and to derive more comparable estimates, we standardized all quantitative predictors to a mean of zero and standard deviation of one. Prior to each analysis, we used variance inflation factors (VIF) to check for collinearity among our explanatory variables. Collinearity was assessed with a cut-off value of 3 [39].

We used the SEM to investigate hypotheses involving the relationships between environmental variables, visitation rates, abundance of flower visitors, and *Bombus* spp., parasite prevalence on pollination. No statistical methodology, including SEM, can by itself demonstrate causation. However, SEM can be used for examining alternative hypotheses and identifying direct and indirect correlations between variables within a defined mechanistic path that incorporates logically plausible causal links; this is potentially a statistically more powerful approach to the analysis of our dataset than other multivariate methods, such as multiple regression, which test all links among all pairs of variables, whether logically plausible or not. Traditional SEM estimation methods assume that all observations are independent, and all variables follow a multivariate normal distribution [40]. In our analyses, we used piecewise SEM that allows fitting generalized linear models to a range of distributions and can account for hierarchy in the data by incorporating hierarchical or nested variables in a mixed model framework [41]. We constructed our *a priori* piecewise SEMs based on previous studies that have tested individual links and hypotheses included in our overall path model [12,20,21,42–45].

We used piecewise SEM [46] for each plant species separately to analyse the direct effects of anthropogenic land-use, local nesting, flower resources and *Bombus* spp. parasite prevalence on pollination service provision or indirect impacts via changes in visitation rates and duration of visits of both all flying insects (Coleoptera; hoverfly; other Diptera; Lepidoptera; bees of the families Andrenidae, Colletidae or Halictidae; *Bombus* spp.; honeybee) and of only bumblebees. Individuals of each experimental plant species nested within a site were treated as a random effect factor.

To explore the potential of multiple factors affecting parasite prevalence among *Bombus* spp., we also performed piecewise structural equation models. We modelled *Crithidia* and *Nosema* prevalence separately as dependent on *Bombus* abundance, using bumblebee age as a covariate, bumblebee morphogroup as a random effect factor, and a binomial error distribution. We hypothesized that local flower richness and anthropogenic land-use can affect parasite prevalence, both directly and indirectly through affecting *Bombus* abundance. To further explore differences in factors affecting parasite prevalence, we conducted separate piecewise SEMs for bumblebee males and females. *Nosema* prevalence was not modelled for females owing to insufficient positive samples (*n* = 2).

**Figure 2.**
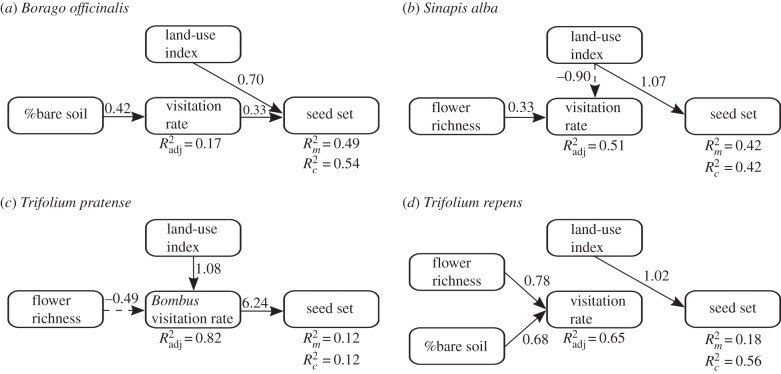
Final path model of anthropogenic land-use and local habitat factors and their relationships with pollination service provision in: (*a*) *Borago officinalis*, (*b*) *Sinapis alba*, (*c*) *Trifolium pratense*, and (*d*) *Trifolium repens*. Black solid arrows show positive and black dashed arrows negative effects derived from piecewise SEM analysis. Unstandardized path coefficients are reported next to the bold arrows and *R*^2^ (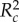 for all factors, 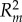 for fixed factors only) values are reported for all response variables. For further information, see electronic supplementary material, table S9.

**Figure 3.**
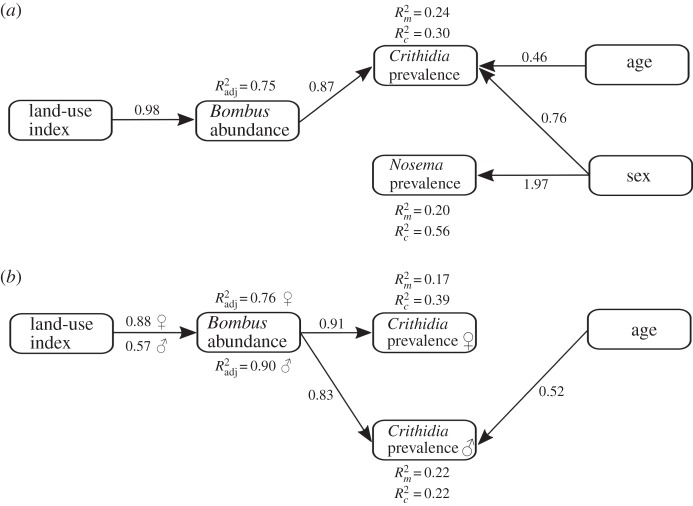
Final path model of anthropogenic land-use and local habitat factors and their relationships with the prevalence of each bumblebee pathogen: (*a*) total and (*b*) separate piecewise SEMs for bumblebee males and females. Black arrows show significant effects derived from piecewise SEM analysis. Unstandardized path coefficients are reported next to the bold arrows and *R*^2^ (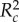 for all factors, 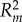 for fixed factors only) values are reported for all response variables.

We performed all mixed models using the package ‘lme4’ v. 1.0-4 [47] and piecewise SEM analyses using the package ‘piecewiseSEM’ [41]. From an overall model based on *a priori* knowledge of interactions with all hypothesized effects, we used a backwards stepwise elimination process based on Akaike Information Criterion modified for small sample sizes (AICc) to remove non-significant pathways. Furthermore, we used the d-separation (d-sep) test to evaluate whether the non-hypothesized independent paths were significant and whether the models could be improved with the inclusion of any of the missing path(s) [46]. Path coefficients and deviance explained were then calculated for each model. We report both conditional (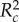, all factors) and marginal (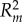, fixed factors only) coefficients of determination for generalized linear-mixed effect models. All correlations between exogenous variables are reported in the supplementary material (electronic supplementary material, table S6), and none of them influenced the final paths presented in figures 2 and 3. All statistical analyses were performed in R v. 3.0.2 [48].

## 3. Results

All plants in our experimental communities produced more seeds per flower unit in the open pollination treatment (*B. officinalis,* 3 ± 0; *S. alba,* 3 ± 0; *T. pratense,* 30 ± 3; *T. repens,* 26 ± 4) compared with the control plants in the glasshouse (*B. officinalis,* 0 ± 0; *S. alba,* 0 ± 0; *T. pratense*, no seeds; *T. repens,* no seeds; Mann–Whitney *U*-test, *p* < 0.05), demonstrating their need for insect visitation to set seed.

### (a) Factors affecting pollination service provision

Visitation rate and total visit duration were strongly correlated (*B. officinalis*, *r* = 0.78, *p* < 0.001; *S. alba*, *r* = 0.72, *p* < 0.001; *T. pratense*, *r* = 0.62, *p* < 0.001; *T. repens*, *r* = 0.74, *p* < 0.001). Therefore, we ran separate analyses with each variable. Here we present the results for visitation rate (for the visit duration results, see electronic supplementary material, table S7).

For piecewise SEMs relating environmental variables, visitation rates, and *Bombus* spp., parasite prevalence on pollination, the stepwise model selection process yielded a final path model well supported by the data (*B. officinalis*: *χ*^2^ = 4.31, d.f. = 4, *p* = 0.365; *S. alba*: *χ*^2^ = 3.36, d.f. = 4, *p* = 0.499; *T. pratense*: *χ*^2^ = 1.78, d.f. = 4, *p* = 0.77; *T. repens*: *χ*^2^ = 5.95, d.f. = 4, *p* = 0.203). Owing to substantial reduction in model fit, the final SEM for our experimental plant species did not include *Crithidia* and *Nosema* prevalence variables. Thus, *Bombus* spp. parasitism was not significantly related to pollination success of our experimental plants.

*Borago officinalis* plants were mainly visited by honeybees (41%, 48 interactions), halictid bees (17%, 21 interactions), and bumblebees (15%, 18 interactions; see supplementary material, table S8). The overall visitation rate of *B. officinalis* was only related to the percentage of bare soil (*p* = 0.005, 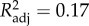; figure 2*a* and see electronic supplementary material, table S9*a*), whereas seed set was not directly related to overall visitation rates (*p* = 0.07; figure 2*a* and see electronic supplementary material, table S9a). *Borago officinalis* plants produced more seeds with increasing urbanization (*p* = 0.003, 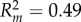, 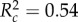; figure 2*a* and see electronic supplementary material, table S9*a*).

*Sinapis alba* plants were mainly visited by hoverflies (75%, 135 interactions) and halictid bees (15%, 28 interactions; see electronic supplementary material, table S8). Overall visitation rates to *S. alba* flowers were positively associated with local flower richness and negatively by increasing urbanization (*p* = 0.05, *p* < 0.001; 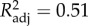, respectively, figure 1*b* and see electronic supplementary material, table S9*b*). However, overall visitation rates were not directly related to *S. alba* seed set (figure 2*b*). Pollination success of *S. alba* also increased in more urban compared with more agricultural areas (*p* = 0.004, 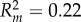, 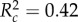; figure 2*b* and see electronic supplementary material, table S9*b*).

*Trifolium pratense* plants were mainly visited by bumblebees (53%, 46 interactions) and butterflies (41%, 36 interactions; see electronic supplementary material, table S8). Overall, *Bombus* visitation rates were positively related to the pollination success of *T. pratense* plants (*p* = 0.04, 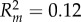, 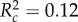; figure 2*c* and see electronic supplementary material, table S9*c*). An increased level of urbanization was indirectly, positively related to seed set via its positive association with bumblebee visitation rates to *T. pratense* plants (*p* < 0.001; 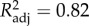; figure 2*c* and see electronic supplementary material, table S9*c*). Yet we also found a negative association between local flower richness and bumblebee visitation rates to *T. pratense* plants (*p* < 0.001; 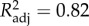; figure 2*c* and see electronic supplementary material, table S9*c*).

*Trifolium repens* plants were mainly visited by bumblebees (30%, 15 interactions), butterflies (14%, seven interactions), and halictid bees (12%, six interactions; see electronic supplementary material, table S8). Both local flower richness and percentage of bare soil were positively related to overall visitation rates to *T. repens* plants (*p* < 0.001 and *p* < 0.001; 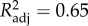; respectively, figure 2*d* and see electronic supplementary material, table S9*d*). However, the direct relationship between overall visitation rates and *T. repens* seed set was minor because it lay below the statistical threshold for inclusion in our final SEM for this plant species (figure 2*d*). The degree of urbanization nevertheless was significantly positively related to the seed set of *T. repens* plants (*p* = 0.04, 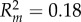, 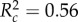; figure 2*d* and see electronic supplementary material, table S9*d*).

### (b) Factors affecting pathogen prevalence among bumblebees

Of the 314 bumblebees sampled, 19% (range 0–37% per site) were infected with *Crithidia*, including 24% of males (0–43% per site) and 13% of females (0–32% per site). *Crithidia* prevalence was lower in *Bombus pascuorum* (7%) than in *Bombus terrestris*/*lucorum* (26%, *χ*^2^ = 12.57, *p* = 0.001) and *Bombus lapidarius/soroeensis proteus* (22%, *χ*^2^ = 8.82, *p* = 0.006) morphogroups. *Nosema bombi* infected on average 6% of all sampled bees (range 0–14% per site), 11% of males (0–18% per site), and 1% of females (0–10% per site). *Nosema* prevalence was higher in *Bombus lapidarius/soroeensis proteus* (14%) than in *Bombus terrestris*/*lucorum* (2%, *χ*^2^ = 10.99, *p* = 0.002) and *Bombus pascuorum* (0%, *χ*^2^ = 18.56, *p* < 0.001).

For piecewise SEMs exploring the potential of multiple factors affecting parasite prevalence among *Bombus* spp., the stepwise model selection process yielded a final path model well supported by the data (pathogen prevalence: *χ*^2^ = 8.53, d.f. = 8, *p* = 0.384; *Crithidia* prevalence in female hosts: *χ*^2^ = 1.46, d.f. = 4, *p* = 0.844; *Crithidia* prevalence in male hosts: *χ*^2^ = 2.14, d.f. = 4, *p* = 0.711). The final model for *Nosema* in males resulted in a poor fit with no significant paths.

**Figure 4.**
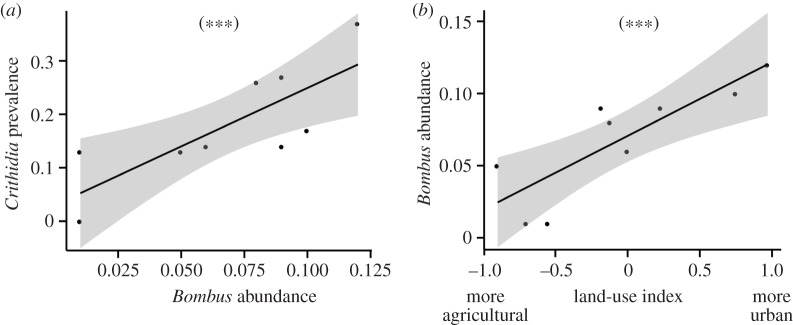
Relationships between (*a*) *Crithidia* prevalence (proportion of bees infected) and *Bombus* spp. abundance, and (*b*) *Bombus* spp. abundance and land-use index. Plotted lines show predicted relationship and the shaded areas indicate the 95% confidence intervals: ***p < 0.001.

There was a positive relationship between *Bombus* abundance and the prevalence of *Crithidia*, both in the entire dataset (*p* < 0.001, figures 3*a* and 4*a*) and when analysed separately by host gender (females: *p* = 0.002; males: *p* < 0.001; figure 3*b*). *Crithidia* parasitism was significantly associated with host gender (*p* = 0.03; figure 3*a*), with a higher prevalence in male bumblebees. The degree of urbanization was indirectly but positively related to *Crithidia* prevalence, via its positive relationship with *Bombus* abundance (*p* < 0.001, figures 3 and 4*b*; *p* < 0.001). Only in host males was host age positively related with *Crithidia* prevalence (*p* = 0.006, figure 3*b*). Surprisingly, we did not find a significant association in the prevalence of *Crithidia* between males and females across sites (Pearson correlation, *r* = 0.43, *p* = 0.27).

The prevalence of *Nosema* was significantly associated only with host gender, with higher prevalence in males (*p* < 0.001; figure 3*a*). *Nosema* prevalence was not related to *Bombus* abundance (*p* = 0.27; figure 3*a*).

### (c) Interacting effects of urbanization and *Bombus* spp., parasitism on pollination service provision

As our results have shown, there was a direct (for *B. officinalis*, *S. alba*, and *T. repens*) and indirect positive relationship between urbanization and pollination success, mediated through elevated overall insect visitation rates (for *B. officinalis*) or elevated *Bombus* spp. only visitation rates (for *T. pratense*; figure 2). Yet, across our land-use gradient, we found indirect positive relationships between urbanization and *Crithidia* prevalence, mediated through increased *Bombus* abundance (figure 3). Thus, urban areas in our study were strongly and positively (though indirectly) related with pollination success through increased overall and *Bombus* spp. visitation rates, despite increased *Crithidia* prevalence with urbanization, which itself was mediated by *Bombus* abundance.

## 4. Discussion

We found that both bumblebee abundance and pollination to wild flowers increased in urban versus rural agricultural sites. Although bumblebee parasitism also increased with host abundance, pollinator parasitism did not have any discernible impact on pollination service provision, even for the bumblebee-dependent species, *T. pratense*. We also found that the availability of nesting resources for ground-nesting bees (Andrenidae, Colletidae, Halictidae) and local flower richness were independently and positively related to visitation rates and pollination of our experimental plant communities.

Seed set of pollinator-dependent self-incompatible plants could be affected both directly and indirectly by a variety of factors. Direct effects occur owing to the lack of compatible pollen donor plants in the vicinity and absence of pollinators (pollinator visit ‘quality’ and ‘quantity’, respectively), and indirect by local flower richness and abundance that themselves influence pollinator visitation rates [49]. In our field experiments, we found that an increase in flower visitation rates was not always directly associated with increased seed set (for *B. officinalis*, *S. alba*, and *T. repens*). This might be due to insufficient sampling, introducing noise to our dataset. Alternatively, or in addition, it may indicate the importance not only of the availability of pollinators in the vicinity to ensure pollination but also of a potential role of the quality of those interactions in terms of the number of compatible, viable pollen grains deposited on stigmata [45]. Increasing nesting resources for ground-nesting bees and flower richness were correlated with overall higher visitation rates by all flower visitors, further emphasizing the importance of local habitat quality for pollinator communities and thus for potential pollination success of wild and crop plants. Finally, our results revealed a positive relationship between urbanization and seed set, highlighting the importance of surrounding land cover in impacting plant–flower visitor interactions that could potentially reflect the increased nesting and food resources for pollinators, especially bees, in moderately urbanized areas (electronic supplementary material, table S6).

To investigate the possible indirect effects of parasitism on pollination, we studied bumblebees and their associated parasites. Within our study, we focused on two important bumblebee parasites that are transmitted horizontally via the oral–faecal route: *Crithidia* and *N. bombi* [50]. Similar to other studies, *Crithidia* was found to be more abundant compared with *N. bombi* [21,43,51]. Furthermore, in our study, prevalence of both parasites was higher in males compared with females. It has been suggested that these higher infection rates may be the result of either life-history differences between the two sexes, with males investing less in immune defence [52], or because infected workers are less likely to leave the nest to forage [51,53]. They may, though, simply reflect a seasonal difference; pathogen prevalence is higher towards the end of summer, a time when males are relatively more abundant. Surprisingly, our results showed no correlation between *Crithidia* prevalence in males and females. This could reflect differences in their dispersal and foraging behaviours, exposure to different communities of pathogen propagules at floral sites of transmission, or their susceptibility to these pathogens. As recent studies highlight the potential role of *Bombus* males as pollinators [54,55], our findings could possibly also translate into differences in the effects of parasitism on the pollination service provided by each sex.

Across host–parasite systems, parasite prevalence is influenced by host population size, and vice versa [56]. We found a positive association between the prevalence of *Crithidia* and overall *Bombus* abundance; the prevalence of *Crithidia* and the abundance of bumblebees increased in more urbanized areas compared with agricultural. These findings suggest that a high prevalence of bumblebee parasites in urban areas could simply reflect the presence of more abundant host populations and greater rates of pathogen transmission [57]. Yet, our results showed no association between *Bombus* parasites studied and seed set produced by our focal plants, even for the bumblebee-dependent species, *T. pratense*. We hypothesize that higher abundances of bumblebees in urban areas resulted in higher success of pollination, which more than compensated for the putative increase in transmission and prevalence of *Crithidia* owing to increasing host abundance.

## 5. Conclusion

Moderate urbanization at the landscape scale and availability of both nesting and flower resources at the local scale positively influenced pollinators and pollination. At the same time, prevalence of parasites in bumblebees, such as *Crithidia*, was higher in urbanized areas compared with agricultural areas, a relationship mediated by elevated *Bombus* abundance. However, we did not find evidence for top-down negative effects of pollinator parasitism on pollination.

## Supplementary Material

Supplementary material

## Supplementary Material

Datasets

## Data accessibility

The datasets used in analyses are available as electronic supplementary material.
