# Supplementary material for: Pollination services enhanced with urbanization despite increasing pollinator parasitism
Source: Proc Biol Sci. 2016 Jun 29;283(1833):20160561. doi: 10.1098/rspb.2016.0561 (PMC4936033; doi:10.1098/rspb.2016.0561)
Supplement: Supplementary material [file rspb20160561supp1.docx]

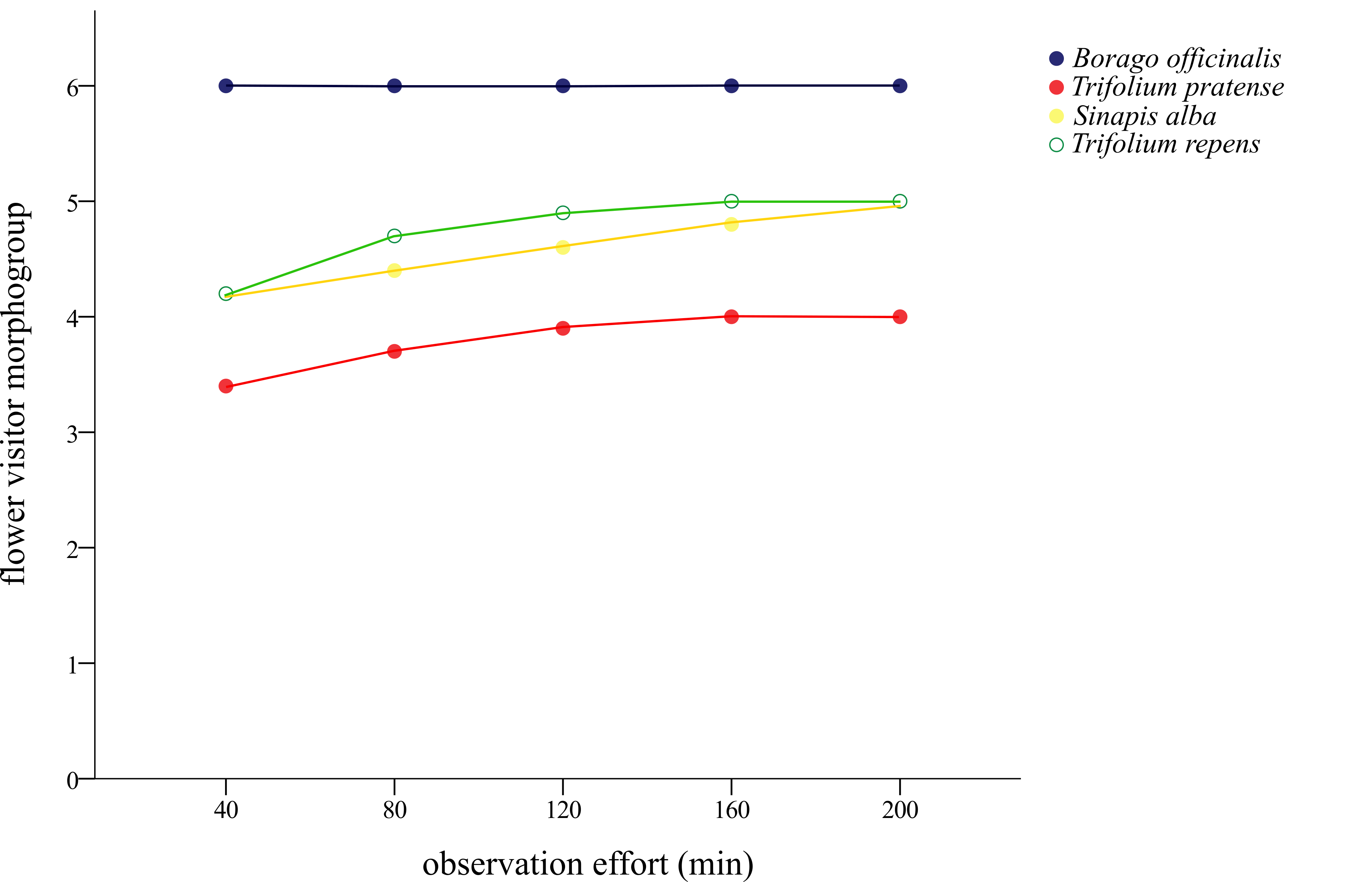


Fig. S1. Flower visitor morphogroup accumulation curves (Mao Tau) as a function of observation time (in minutes) across all nine sites for each of the four phytometer plant species listed in the key.

Table S1. Coordinates of field sites used in our study and land-use index based on the proportional area of each land class within a 750m radius (see main text, Methods). The index varies from -1 (entirely agricultural) to +1 (entirely urban); these two major land-use classes made up >75% of land use across all sites and > 40% within every site.

| Site | Latitude | Longitude | Land-use index  (-1 to +1) | Weather  first round (July 2013, for *S. alba* and *B. officinalis*) | | Weather  second round (August 2013, for *Trifolium* spp.) | |
| --- | --- | --- | --- | --- | --- | --- | --- |
|  |  |  |  | Temperature (°C) (morning/afternoon) | Wind speed (ms^-1^)  (morning/afternoon) | Temperature (°C) (morning/afternoon) | Wind speed (m/s)  (morning/afternoon) |
| Kröllwitz | 51°30'29.69"N | 11°55'48.31"E | 0.22 | 21°C/24°C | 0.7 ms^-1^/0.3 ms^-1^ | 31°C/27°C | 0.5 ms^-1^/1.2 ms^-1^ |
| Botanical  Gardens Halle | 51°29'22.76"N | 11°57'40.85"E | 0.97 | 25°C/27°C | 0.4 ms^-1^/0.4 ms^-1^ | 29°C/25°C | 0.3 ms^-1^/0.4 ms^-1^ |
| Thüringer Bhf | 51°27'44.35"N | 11°59'24.85"E | 0.74 | 25°C/28°C | 0.4 ms^-1^/0.4 ms^-1^ | 29°C/32°C | 0.6 ms^-1^ /0.8 ms^-1^ |
| Bad Lauchstädt | 51°23'28.02"N | 11°52'44.09"E | -0.11 | 24°C/27°C | 0.4 ms^-1^/0.6 ms^-1^ | 26°C/22°C | 1.5 ms^-1^ /1.5 ms^-1^ |
| Teutschenthal | 51°25'13.73"N | 11°50'06.32"E | -0.90 | 20°C/27°C | 0.5 ms^-1^/1.4 ms^-1^ | 25°C/29°C | 0.9 ms^-1^/1.3 ms^-1^ |
| Amsdorf | 51°26'42.90"N | 11°42'14.66"E | 0 | 26°C/28°C | 0.5 ms^-1^/0.5 ms^-1^ | 27°C/31°C | 1.1 ms^-1^/0.3 ms^-1^ |
| Salziger See | 51°28'51.00"N | 11°40'56.00"E | -0.55 | 24°C/29°C | 0.8 ms^-1^/1.5 ms^-1^ | 26°C/30°C | 0.4 ms^-1^/0.3 ms^-1^ |
| Klobikau | 51°20'25.15"N | 11°50'44.20"E | -0.18 | 24°C/30°C | 0.3 ms^-1^/0.2 ms^-1^ | 21°C/25°C | 1.4 ms^-1^/0.7 ms^-1^ |
| Friedeburg | 51°37'19.00"N | 11°43'26.00"E | -0.70 | 24°C/29°C | 0.5 ms^-1^/0.4 ms^-1^ | 27°C/28°C | 1.2 ms^-1^ /1.3 ms^-1^ |

| Fclass | Description |  |
| --- | --- | --- |
| Forest | A forest or woodland |  |
| Park | A park |  |
| Residential | A residential area |  |
| Industrial | An industrial area |  |
| Arable land | Agricultural land (farms and areas where crops are grown) |  |
| Allotments | An area with small private gardens |  |
| Meadow | A meadow, possibly used for grazing cattle |  |
| Commercial | A commercial area |  |
| Nature reserve | A nature reserve |  |
| Retail | An area mainly used by shops |  |
| Quarry | A quarry |  |
| Orchard | An area used for growing fruit-bearing trees |  |
| Grass | Semi-natural grassland |  |
| Scrub | Area of scrub vegetation |  |

Table S2. Land-cover types provided by land cover data obtained from Geofabrik GmbH.

Table S3. Pearson’s correlation coefficients (*r_s_*) of the relationship between abundance of flower visitors and landscape diversity with increasing area (given as radius in metres) from the centre of each site.

| Radius | 500 m | 750 m | 1000 m | 1500 m | 2000 m |
| --- | --- | --- | --- | --- | --- |
| *r_s_* | 0.132 | **0.254** | 0.215 | 0.083 | 0.0002 |

Table S4. Numbers of bumble bees (*Bombus* spp.) by sampling date, morphogroup and sex at each site in 2013.

|  |  | Females | | |  | Males | | |  |  |
| --- | --- | --- | --- | --- | --- | --- | --- | --- | --- | --- |
| Site | Dates | *Bombus*  *pascuorum* | *Bombus*  *terrestris* | *Bombus*  *lapidarius* | Total  females | *Bombus*  *pascuorum* | *Bombus*  *terrestris* | *Bombus*  *lapidarius* | Total  males | Total |
| Kröllwitz | 01-04 Aug. | 11 | - | 8 | 19 | 2 | - | 7 | 9 | 28 |
| Botanical Gardens Halle | 01-04 Aug. | 15 | 5 | 5 | 35 | 7 | 7 | 7 | 21 | 46 |
| Thüringer Bahnhof | 01-04 Aug. | 7 | 7 | 7 | 21 | 2 | 7 | 8 | 17 | 38 |
| Bad Lauchstädt | 10-14 Aug. | 16 | 2 | 2 | 20 | 7 | 7 | 7 | 21 | 41 |
| Teutschenthal | 10-14 Aug. | - | 6 | 10 | 16 | 7 | - | 7 | 14 | 30 |
| Amsdorf | 15-19 Aug. | - | 5 | 9 | 14 | - | 7 | 7 | 14 | 28 |
| Salziger See | 15-19 Aug. | 7 | 7 | 7 | 21 | 2 | 7 | 4 | 13 | 34 |
| Klobikau | 10-14 Aug. | 7 | - | - | 7 | 7 | 7 | 7 | 21 | 28 |
| Friedeburg | 15-19 Aug. | 7 | 7 | 7 | 21 | 6 | 7 | 7 | 20 | 41 |

| Variable | Mantel test | |
| --- | --- | --- |
|  | *R^2^* | *p* |
| a) Seed set |  |  |
| *Borago officinalis* | 0.007 | 0.292 |
| *Sinapis alba* | 0.008 | 0.579 |
| *Trifolium pratense* | 0.078 | 0.092 |
| *Trifolium repens* | 0.006 | 0.617 |
|  |  |  |
| b) Visitation rate |  |  |
| *Borago officinalis* | 0.020 | 0.793 |
| *Sinapis alba* | 0.407 | 0.061 |
| *Trifolium pratense* | 0.195 | 0.080 |
| *Trifolium repens* | <0.001 | 0.431 |
|  |  |  |
| c) Pathogen prevalence |  |  |
| *Crithidia* | 0.056 | 0.122 |
| *Nosema* | <0.001 | 0.424 |

Table S5. Summary of Mantel tests between (a) seed set, (b) visitation rate, or (c) pathogen prevalence and geographic distance between sites to test for spatial autocorrelation. Results include Mantel’s correlation *R^2^* and simulated *p* value. Values were derived from 10,000 permutations.

Table S6. Pearson’s correlation coefficients (*r_s_*) of the relationship between all exogenous variables used in our analysis (below diagonal) and *p* values (above diagonal).

| Variable | Land-use index | % Bare soil | Local flower richness |
| --- | --- | --- | --- |
| Land-use index | - | 0.233 | 0.049 |
| % Bare soil | 0.442 | - | 0.714 |
| Local flower richness | 0.667 | 0.142 | - |

Table S7. Table of path coefficients from the final best-fit piecewise SEM models explaining the effects of local habitat, land-use index and parasite prevalence on the mean number of seeds produced by (a) *Borago officinalis*, (b) *Sinapis alba*, (c) *Trifolium pratense* and (d) *Trifolium repens*, either directly or via their effect on total visit duration (as opposed to vitiation rates). Parasite prevalence did not reach significance in any model. We used the AICc method for model selection. ‘Visit duration’ includes all (Coleopteran, hoverfly, other Dipteran, Lepidopteran, *Bombus* spp. and honey bee) visits whereas ‘*Bombus* visit duration’ includes only *Bombus* spp. visits.

| Model | Regression weights  (given as y upon x) | | Estimate | | S.E. | *p* | *R^2^* |
| --- | --- | --- | --- | --- | --- | --- | --- |
| (a) *B. officinalis* |  | |  | |  |  |  |
| Visit duration | 🡨 | Flower richness | | -0.16 | 0.15 | 0.2 | *R^2^*_m_=0.02 |
| Seed set | 🡨 | Land-use index | | 0.76 | 0.14 | 0.001** | *R^2^*_m_=0.51  *R^2^_c_*=0.54 |
| Seed set | 🡨 | Visit duration | | 0.37 | 0.14 | 0.04* |  |
| (b) *S. alba* |  | |  | |  |  |  |
| Visit duration | 🡨 | Flower richness | | 0.82 | 0.16 | <0.001** | *R^2^*_m_=0.48 |
| Visit duration | 🡨 | Land-use index | | -0.89 | 0.17 | <0.001*** |  |
| Seed set | 🡨 | Land-use index | | 1.02 | 0.23 | 0.01* | *R^2^*_m_=0.42  *R^2^_c_*=0.42 |
| Seed set | 🡨 | Visit duration | | -0.14 | 0.23 | 0.5 |  |
| (c) *T. pratense* |  | | |  |  |  |  |
| *Bombus* visit duration | 🡨 | Land-use index | | 0.76 | 0.08 | <0.001*** | *R^2^*_m_=0.81 |
| *Bombus* visit duration | 🡨 | Flower richness | | 0.22 | 0.08 | 0.008** |  |
| Seed set | 🡨 | *Bombus* visit duration | | 5.55 | 2.58 | 0.06 | *R^2^*_m_=0.09  *R^2^_c_*=0.10 |
| (b) *T. repens* |  | | |  |  |  |  |
| Visit duration | 🡨 | Flower richness | | 0.30 | 0.15 | 0.05* | *R^2^*_m_=0.10 |
| Seed set | 🡨 | Land-use index | | 1.02 | 0.51 | 0.04* | *R^2^*_m_=0.31  *R^2^_c_*=0.90 |

*, *p*<0.05; **, *p<*0.01; ***, *p*<0.001

Table S8. Overall number of visits per pollinator morphogroup recorded on *Borago officinalis*, *Sinapis alba*, *Trifolium pratense* and *Trifolium repens* plants.

| Plant species | Syrphidae | Bumblebees | Coleoptera | Lepidoptera | *Apis mellifera* | Halictidae | Andrenidae/Colletidae | Total |
| --- | --- | --- | --- | --- | --- | --- | --- | --- |
| *Borago officinalis* | 8 | 18 | 0 | 3 | 48 | 21 | 18 | 116 |
| *Sinapis alba* | 135 | 1 | 2 | 1 | 0 | 28 | 11 | 178 |
| *Trifolium pratense* | 0 | 46 | 0 | 36 | 0 | 1 | 4 | 87 |
| *Trifolium repens* | 0 | 15 | 0 | 7 | 1 | 6 | 7 | 49 |

Table S9. Table of path coefficients from the final best-fit piecewise SEM models explaining the effects of local habitat, our anthropogenic land-use index and *Bombus* parasite prevalence on the mean number of seeds produced by (a), *Borago officinalis* (b) *Sinapis alba,* (c) *Trifolium pratense* and (d) *Trifolium repens*, either directly or via visitation rates (as opposed to visit duration). Parasite prevalence did not reach significance in any model. We used the AICc method for model selection. ‘Visitation rate’ includes all (Coleopteran, hoverfly, other Dipteran, Lepidopteran, *Bombus* spp., and honey bee) visits whereas ‘*Bombus* visitation rate’ includes only *Bombus* spp. visits.

| Model | Regression weights | | | Estimate | | | | S.E. | *p* | | |
| --- | --- | --- | --- | --- | --- | --- | --- | --- | --- | --- | --- |
| (a) *B. officinalis* |  | | |  | | | |  |  | | |
| Visitation rate | | 🡨 | % Bare soil | | 0.42 | | | 0.14 | | 0.005** |  |
| Seed set | | 🡨 | Land-use index | | 0.70 | | | 0.15 | | 0.003** |  |
| Seed set | | 🡨 | Visitation rate | | 0.33 | | | 0.16 | | 0.07 |  |
| (b) *S. alba* |  | | |  | | | |  |  | | |
| Visitation rate | | 🡨 | Flower richness | | | 0.33 | | 0.17 | | 0.05* |  |
| Visitation rate | | 🡨 | Land-use index | | | -0.90 | | 0.17 | | <0.001*** |  |
| Seed set | | 🡨 | Land-use index | | | 1.07 | | 0.22 | | 0.004** |  |
| (c) *T. pratense* |  | | |  | | |  | |  | | |
| *Bombus* visitation rate | | 🡨 | Flower richness | | | -0.49 | | 0.07 | | <0.001*** |  |
| *Bombus* visitation rate | | 🡨 | Land-use index | | | 1.08 | | 0.07 | | <0.001*** |  |
| Seed set | | 🡨 | *Bombus* visitation rate | | | 6.24 | | 2.55 | | 0.04* |  |
| (b) *T. repens* |  | | |  | | | |  |  | | |
| Visitation rate | | 🡨 | Flower richness | | | 0.78 | | 0.12 | | <0.001*** |  |
| Visitation rate | | 🡨 | % Bare soil | | | 0.68 | | 0.10 | | <0.001*** |  |
| Seed set | | 🡨 | Land-use index | | | 1.02 | | 0.51 | | 0.04* |  |

*, *p*<0.05; **, *p*<0.01; ***, *p*<0.001
