# Supplementary material for: Pollination services enhanced with urbanization despite increasing pollinator parasitism
Source: Proc Biol Sci. 2016 Jun 29;283(1833):20160561. doi: 10.1098/rspb.2016.0561 (PMC4936033; doi:10.1098/rspb.2016.0561)
Supplement: Datasets [file rspb20160561supp2.pdf]

Supplementary material: Datasets used in analyses

Table 1. Pollination experiments (a) *B.officinalis* , (b) *Sinapis alba* , (c) *Trifolium pratense* and (d) *Trifolium repens*

(a) *Borago officinalis*

Mean number of seed set per flower	Plant	Land_Use_index	BareSoil	FlowerRichness	Lat	Long	Visitation_Rate	*Crithidia*	*Nosema*	Duration_Visit	Sites
3.52	5	0.97	26.90	17.00	51.49	11.96	0.12	0.37	0.02	56.00	Botanical Gardens Halle
4.81	4	0.97	26.90	17.00	51.49	11.96	0.12	0.37	0.02	56.00	Botanical Gardens Halle
3.71	3	0.97	26.90	17.00	51.49	11.96	0.12	0.37	0.02	56.00	Botanical Gardens Halle
3.67	2	0.97	26.90	17.00	51.49	11.96	0.12	0.37	0.02	56.00	Botanical Gardens Halle
3.05	5	0.75	11.50	9.00	51.46	11.99	0.10	0.16	0.08	28.20	Thüringer Bahn
3.76	4	0.75	11.50	9.00	51.46	11.99	0.10	0.16	0.08	28.20	Thüringer Bahn
4.52	3	0.75	11.50	9.00	51.46	11.99	0.10	0.16	0.08	28.20	Thüringer Bahn
5.00	2	0.75	11.50	9.00	51.46	11.99	0.10	0.16	0.08	28.20	Thüringer Bahn
2.43	1	0.75	11.50	9.00	51.46	11.99	0.10	0.16	0.08	28.20	Thüringer Bahn
3.52	5	0.23	15.10	10.00	51.51	11.93	0.20	0.25	0.14	113.60	Kröllwitz
3.57	4	0.23	15.10	10.00	51.51	11.93	0.20	0.25	0.14	113.60	Kröllwitz
3.57	3	0.23	15.10	10.00	51.51	11.93	0.20	0.25	0.14	113.60	Kröllwitz
2.48	2	0.23	15.10	10.00	51.51	11.93	0.20	0.25	0.14	113.60	Kröllwitz
3.95	1	0.23	15.10	10.00	51.51	11.93	0.20	0.25	0.14	113.60	Kröllwitz
3.67	1	-0.12	17.60	10.00	51.39	11.88	0.24	0.27	0.07	211.00	Bad Lauchstädt
4.90	2	-0.12	17.60	10.00	51.39	11.88	0.24	0.27	0.07	211.00	Bad Lauchstädt
3.86	3	-0.12	17.60	10.00	51.39	11.88	0.24	0.27	0.07	211.00	Bad Lauchstädt
2.43	4	-0.12	17.60	10.00	51.39	11.88	0.24	0.27	0.07	211.00	Bad Lauchstädt
3.00	5	-0.12	17.60	10.00	51.39	11.88	0.24	0.27	0.07	211.00	Bad Lauchstädt
2.95	2	-0.90	15.60	5.00	51.42	11.84	0.06	0.13	0.07	19.75	Teutschenthal
0.71	3	-0.90	15.60	5.00	51.42	11.84	0.06	0.13	0.07	19.75	Teutschenthal
2.38	4	-0.90	15.60	5.00	51.42	11.84	0.06	0.13	0.07	19.75	Teutschenthal
1.81	1	-0.70	4.20	7.00	51.62	11.72	0.09	0.00	0.07	80.20	Friedeburg
0.52	2	-0.70	4.20	7.00	51.62	11.72	0.09	0.00	0.07	80.20	Friedeburg
0.33	3	-0.70	4.20	7.00	51.62	11.72	0.09	0.00	0.07	80.20	Friedeburg
1.38	4	-0.70	4.20	7.00	51.62	11.72	0.09	0.00	0.07	80.20	Friedeburg
2.43	5	-0.70	4.20	7.00	51.62	11.72	0.09	0.00	0.07	80.20	Friedeburg
2.57	1	-0.55	5.30	11.00	51.48	11.68	0.09	0.15	0.00	20.80	Salziger See
3.00	2	-0.55	5.30	11.00	51.48	11.68	0.09	0.15	0.00	20.80	Salziger See
1.86	3	-0.55	5.30	11.00	51.48	11.68	0.09	0.15	0.00	20.80	Salziger See
0.95	4	-0.55	5.30	11.00	51.48	11.68	0.09	0.15	0.00	20.80	Salziger See
0.95	5	-0.55	5.30	11.00	51.48	11.68	0.09	0.15	0.00	20.80	Salziger See
2.52	1	-0.18	13.70	11.00	51.34	11.85	0.20	0.15	0.00	40.40	Klobikau
1.67	2	-0.18	13.70	11.00	51.34	11.85	0.20	0.15	0.00	40.40	Klobikau
2.57	3	-0.18	13.70	11.00	51.34	11.85	0.20	0.15	0.00	40.40	Klobikau
2.62	4	-0.18	13.70	11.00	51.34	11.85	0.20	0.15	0.00	40.40	Klobikau
2.00	5	-0.18	13.70	11.00	51.34	11.85	0.20	0.15	0.00	40.40	Klobikau
2.71	1	0.00	10.20	15.00	51.45	11.70	0.07	0.15	0.07	19.66	Amsdorf
1.43	2	0.00	10.20	15.00	51.45	11.70	0.07	0.15	0.07	19.66	Amsdorf
1.95	3	0.00	10.20	15.00	51.45	11.70	0.07	0.15	0.07	19.66	Amsdorf
1.81	4	0.00	10.20	15.00	51.45	11.70	0.07	0.15	0.07	19.66	Amsdorf
1.57	5	0.00	10.20	15.00	51.45	11.70	0.07	0.15	0.07	19.66	Amsdorf
2.43	6	0.00	10.20	15.00	51.45	11.70	0.07	0.15	0.07	19.66	Amsdorf

(b) *Sinapis alba*

Mean number of seed set per flower	Plant	Land-use index	BareSoil	FlowerRichness	Visitation_Rate	*Crithidia*	*Nosema*	Duration_visit	Sites
4.68	2	0.97	26.90	17	0.05	0.37	0.02	13.80	Botanical Gardens Halle
3.18	4	0.97	26.90	17	0.05	0.37	0.02	13.80	Botanical Gardens Halle
1.03	5	0.97	26.90	17	0.05	0.37	0.02	13.80	Botanical Gardens Halle
5.95	3	0.97	26.90	17	0.05	0.37	0.02	13.80	Botanical Gardens Halle
4.98	1	0.97	26.90	17	0.05	0.37	0.02	13.80	Botanical Gardens Halle
3.93	2	0.75	11.50	9	0.06	0.16	0.08	40.80	Thüringer Bahn
3.18	1	0.75	11.50	9	0.06	0.16	0.08	40.80	Thüringer Bahn
4.48	3	0.75	11.50	9	0.06	0.16	0.08	40.80	Thüringer Bahn
5.65	4	0.75	11.50	9	0.06	0.16	0.08	40.80	Thüringer Bahn
3.98	5	0.75	11.50	9	0.06	0.16	0.08	40.80	Thüringer Bahn
3.53	1	0.23	15.10	10	0.03	0.25	0.14	7.60	Kröllwitz
5.78	2	0.23	15.10	10	0.03	0.25	0.14	7.60	Kröllwitz
2.18	3	0.23	15.10	10	0.03	0.25	0.14	7.60	Kröllwitz
1.85	4	0.23	15.10	10	0.03	0.25	0.14	7.60	Kröllwitz
2.68	5	0.23	15.10	10	0.03	0.25	0.14	7.60	Kröllwitz
2.80	2	-0.90	15.60	5	0.19	0.13	0.07	62.40	Teutschenthal
2.63	3	-0.90	15.60	5	0.19	0.13	0.07	62.40	Teutschenthal
0.28	1	-0.90	15.60	5	0.19	0.13	0.07	62.40	Teutschenthal
0.13	5	-0.90	15.60	5	0.19	0.13	0.07	62.40	Teutschenthal
0.08	4	-0.90	15.60	5	0.19	0.13	0.07	62.40	Teutschenthal
1.75	1	-0.70	4.20	7	0.39	0.00	0.07	160.80	Friedeburg
2.33	2	-0.70	4.20	7	0.39	0.00	0.07	160.80	Friedeburg

1.15	4	-0.70	4.20	7	0.39	0.00	0.07	160.80	Friedeburg
0.80	5	-0.70	4.20	7	0.39	0.00	0.07	160.80	Friedeburg
0.48	3	-0.70	4.20	7	0.39	0.00	0.07	160.80	Friedeburg
2.00	1	-0.55	5.30	11	0.16	0.15	0.00	175.00	Salziger See
2.83	2	-0.55	5.30	11	0.16	0.15	0.00	175.00	Salziger See
2.40	3	-0.55	5.30	11	0.16	0.15	0.00	175.00	Salziger See
3.60	4	-0.55	5.30	11	0.16	0.15	0.00	175.00	Salziger See
2.00	5	-0.55	5.30	11	0.16	0.15	0.00	175.00	Salziger See
2.05	4	0.00	10.20	15	0.31	0.14	0.07	408.40	Amsdorf
1.58	2	0.00	10.20	15	0.31	0.14	0.07	408.40	Amsdorf
0.95	3	0.00	10.20	15	0.31	0.14	0.07	408.40	Amsdorf
4.15	5	0.00	10.20	15	0.31	0.14	0.07	408.40	Amsdorf

(c) *Trifolium pratense*

Mean number of seeds per inflorecence	Plant	Land_use_index	BareSoil	Flower_Richness	*Crithidia*	*Nosema*	Visit_Rate_*Bombus*	*Bombus* Duration_of_visit	Sites
21.30	1	0.97	21.60	23	0.37	0.02	0.12	197.20	Botanical Gardens Halle
37.75	2	0.97	21.60	23	0.37	0.02	0.12	197.20	Botanical Gardens Halle
37.80	3	0.97	21.60	23	0.37	0.02	0.12	197.20	Botanical Gardens Halle
66.25	4	0.97	21.60	23	0.37	0.02	0.12	197.20	Botanical Gardens Halle
34.25	5	0.97	21.60	23	0.37	0.02	0.12	197.20	Botanical Gardens Halle
33.00	1	0.75	15.50	12	0.16	0.08	0.12	161.80	Thüringer Bahn
50.25	2	0.75	15.50	12	0.16	0.08	0.12	161.80	Thüringer Bahn
56.25	3	0.75	15.50	12	0.16	0.08	0.12	161.80	Thüringer Bahn
32.00	4	0.75	15.50	12	0.16	0.08	0.12	161.80	Thüringer Bahn
26.25	5	0.75	15.50	12	0.16	0.08	0.12	161.80	Thüringer Bahn
59.33	1	0.23	9.80	16	0.25	0.14	0.06	129.00	Kröllwitz
55.00	2	0.23	9.80	16	0.25	0.14	0.06	129.00	Kröllwitz
14.00	3	0.23	9.80	16	0.25	0.14	0.06	129.00	Kröllwitz
16.57	4	0.23	9.80	16	0.25	0.14	0.06	129.00	Kröllwitz
25.63	5	0.23	9.80	16	0.25	0.14	0.06	129.00	Kröllwitz
8.00	1	-0.12	22.70	5	0.27	0.07	0.11	51.60	Bad Lauchstädt
77.00	2	-0.12	22.70	5	0.27	0.07	0.11	51.60	Bad Lauchstädt
60.00	3	-0.12	22.70	5	0.27	0.07	0.11	51.60	Bad Lauchstädt
21.50	4	-0.12	22.70	5	0.27	0.07	0.11	51.60	Bad Lauchstädt
13.50	5	-0.12	22.70	5	0.27	0.07	0.11	51.60	Bad Lauchstädt
27.50	1	-0.90	21.30	8	0.13	0.07	0.00	8.20	Teutschenthal
8.60	2	-0.90	21.30	8	0.13	0.07	0.00	8.20	Teutschenthal
7.40	5	-0.90	21.30	8	0.13	0.07	0.00	8.20	Teutschenthal
15.00	1	-0.70	3.00	15	0.00	0.07	0.01	70.60	Friedeburg
42.00	2	-0.70	3.00	15	0.00	0.07	0.01	70.60	Friedeburg
34.75	3	-0.70	3.00	15	0.00	0.07	0.01	70.60	Friedeburg
14.57	4	-0.70	3.00	15	0.00	0.07	0.01	70.60	Friedeburg
13.25	5	-0.70	3.00	15	0.00	0.07	0.01	70.60	Friedeburg
45.33	1	-0.55	7.30	9	0.15	0.00	0.02	9.60	Salziger See
11.00	2	-0.55	7.30	9	0.15	0.00	0.02	9.60	Salziger See
17.33	3	-0.55	7.30	9	0.15	0.00	0.02	9.60	Salziger See
46.20	4	-0.55	7.30	9	0.15	0.00	0.02	9.60	Salziger See
32.00	5	-0.55	7.30	9	0.15	0.00	0.02	9.60	Salziger See
10.70	1	-0.18	20.60	14	0.14	0.00	0.07	12.40	Klobikau
12.50	2	-0.18	20.60	14	0.14	0.00	0.07	12.40	Klobikau
31.00	3	-0.18	20.60	14	0.14	0.00	0.07	12.40	Klobikau
26.83	4	-0.18	20.60	14	0.14	0.00	0.07	12.40	Klobikau
38.50	5	-0.18	20.60	14	0.14	0.00	0.07	12.40	Klobikau
15.33	1	0.00	6.00	17	0.14	0.07	0.02	71.80	Amsdorf
18.50	2	0.00	6.00	17	0.14	0.07	0.02	71.80	Amsdorf
44.00	3	0.00	6.00	17	0.14	0.07	0.02	71.80	Amsdorf
21.80	4	0.00	6.00	17	0.14	0.07	0.02	71.80	Amsdorf
24.20	5	0.00	6.00	17	0.14	0.07	0.02	71.80	Amsdorf

(d) *Trifolium repens*

Total number of seeds per inflorecence	Plant	Land_use_index	BareSoil	FlowerRichness	*Crithidia*	*Nosema*	Visit_Rate	Duration_visit	Sites
201	1	0.97	21.60	23	0.37	0.02	0.09	53.20	Botanical Gardens Halle
313	2	0.97	21.60	23	0.37	0.02	0.09	53.20	Botanical Gardens Halle
130	3	0.97	21.60	23	0.37	0.02	0.09	53.20	Botanical Gardens Halle
715	4	0.97	21.60	23	0.37	0.02	0.09	53.20	Botanical Gardens Halle
854	5	0.97	21.60	23	0.37	0.02	0.09	53.20	Botanical Gardens Halle
268	1	0.75	15.50	12	0.16	0.08	0.02	6.80	Thüringer Bahn
86	2	0.75	15.50	12	0.16	0.08	0.02	6.80	Thüringer Bahn
0	3	0.75	15.50	12	0.16	0.08	0.02	6.80	Thüringer Bahn
76	4	0.75	15.50	12	0.16	0.08	0.02	6.80	Thüringer Bahn
129	5	0.75	15.50	12	0.16	0.08	0.02	6.80	Thüringer Bahn
122	1	0.23	9.80	16	0.25	0.14	0.08	116.40	Kröllwitz
292	2	0.23	9.80	16	0.25	0.14	0.08	116.40	Kröllwitz
39	3	0.23	9.80	16	0.25	0.14	0.08	116.40	Kröllwitz

37	4	0.23	9.80	16	0.25	0.14	0.08	116.40	Kröllwitz
43	5	0.23	9.80	16	0.25	0.14	0.08	116.40	Kröllwitz
23	1	-0.12	22.70	5	0.27	0.07	0.05	30.40	Bad Lauchstädt
266	2	-0.12	22.70	5	0.27	0.07	0.05	30.40	Bad Lauchstädt
719	3	-0.12	22.70	5	0.27	0.07	0.05	30.40	Bad Lauchstädt
457	4	-0.12	22.70	5	0.27	0.07	0.05	30.40	Bad Lauchstädt
213	5	-0.12	22.70	5	0.27	0.07	0.05	30.40	Bad Lauchstädt
2	1	-0.90	21.30	8	0.13	0.07	0.04	56.80	Teutschenthal
2	2	-0.90	21.30	8	0.13	0.07	0.04	56.80	Teutschenthal
0	3	-0.90	21.30	8	0.13	0.07	0.04	56.80	Teutschenthal
10	1	-0.70	3.00	15	0.00	0.07	0.03	13.60	Friedeburg
129	2	-0.70	3.00	15	0.00	0.07	0.03	13.60	Friedeburg
11	3	-0.70	3.00	15	0.00	0.07	0.03	13.60	Friedeburg
62	4	-0.70	3.00	15	0.00	0.07	0.03	13.60	Friedeburg
118	5	-0.70	3.00	15	0.00	0.07	0.03	13.60	Friedeburg
119	1	-0.55	7.30	9	0.15	0.00	0.00	0.00	Salziger See
66	2	-0.55	7.30	9	0.15	0.00	0.00	0.00	Salziger See
10	3	-0.55	7.30	9	0.15	0.00	0.00	0.00	Salziger See
36	4	-0.55	7.30	9	0.15	0.00	0.00	0.00	Salziger See
77	5	-0.55	7.30	9	0.15	0.00	0.00	0.00	Salziger See
150	1	-0.18	20.60	14	0.14	0.00	0.08	40.40	Klobikau
118	2	-0.18	20.60	14	0.14	0.00	0.08	40.40	Klobikau
219	3	-0.18	20.60	14	0.14	0.00	0.08	40.40	Klobikau
112	4	-0.18	20.60	14	0.14	0.00	0.08	40.40	Klobikau
0	5	-0.18	20.60	14	0.14	0.00	0.08	40.40	Klobikau
10	1	0.00	6.00	17	0.14	0.07	0.04	5.80	Amsdorf
0	3	0.00	6.00	17	0.14	0.07	0.04	5.80	Amsdorf
0	4	0.00	6.00	17	0.14	0.07	0.04	5.80	Amsdorf

Table 2. Pathogen prevalence among bumblebees

SamplesID	Sex	*Crithidia*	*Nosema*	Morphogroup	Land_use_index	Flower_richness	Bombus_Abundace	BareSoil	Age
2BPBOT1	F	NO	NO	BP	0.97	20	0.12	24.25	2
2BPBOT2	F	NO	NO	BP	0.97	20	0.12	24.25	2
2BPBOT3	F	NO	NO	BP	0.97	20	0.12	24.25	5
2BPBOT4	F	NO	NO	BP	0.97	20	0.12	24.25	3
2BPBOT5	F	NO	NO	BP	0.97	20	0.12	24.25	4
2BPBOT6	F	NO	NO	BP	0.97	20	0.12	24.25	3
2BPBOT7	F	NO	NO	BP	0.97	20	0.12	24.25	1
2BPBOT8	F	NO	NO	BP	0.97	20	0.12	24.25	1
2BPBOT9	F	NO	NO	BP	0.97	20	0.12	24.25	2
2BPBOT10	F	NO	NO	BP	0.97	20	0.12	24.25	5
2BPBOT11	F	NO	NO	BP	0.97	20	0.12	24.25	1
2BPBOT12	F	NO	NO	BP	0.97	20	0.12	24.25	2
2BPBOT13	F	NO	NO	BP	0.97	20	0.12	24.25	3
2BPBOT14	F	NO	NO	BP	0.97	20	0.12	24.25	4
2BPBOT15	F	NO	NO	BP	0.97	20	0.12	24.25	4
2BTBOT1	F	YES	NO	BT	0.97	20	0.12	24.25	3
2BTBOT2	F	NO	NO	BT	0.97	20	0.12	24.25	5
2BTBOT3	F	YES	NO	BT	0.97	20	0.12	24.25	5
2BTBOT4	F	YES	NO	BT	0.97	20	0.12	24.25	3
2BTBOT5	F	YES	NO	BT	0.97	20	0.12	24.25	3
2BLBOT1	F	NO	NO	BL	0.97	20	0.12	24.25	3
2BLBOT2	F	YES	NO	BL	0.97	20	0.12	24.25	3
2BLBOT3	F	YES	NO	BL	0.97	20	0.12	24.25	3
2BLBOT4	F	YES	NO	BL	0.97	20	0.12	24.25	4
2BLBOT5	F	YES	NO	BL	0.97	20	0.12	24.25	3
2BLPR01	F	NO	NO	BL	0.75	10.5	0.1	13.5	2
2BLPR02	F	YES	NO	BL	0.75	10.5	0.1	13.5	5
2BLPR03	F	NO	NO	BL	0.75	10.5	0.1	13.5	3
2BLPR04	F	YES	NO	BL	0.75	10.5	0.1	13.5	5
2BLPR05	F	NO	NO	BL	0.75	10.5	0.1	13.5	3
2BLPR06	F	NO	NO	BL	0.75	10.5	0.1	13.5	3
2BLPR07	F	NO	NO	BL	0.75	10.5	0.1	13.5	4
2BPPR01	F	NO	NO	BP	0.75	10.5	0.1	13.5	1
2BPPR02	F	NO	NO	BP	0.75	10.5	0.1	13.5	1
2BPPR03	F	NO	NO	BP	0.75	10.5	0.1	13.5	2
2BPPR04	F	NO	NO	BP	0.75	10.5	0.1	13.5	2
2BPPR05	F	NO	NO	BP	0.75	10.5	0.1	13.5	2
2BPPR06	F	NO	NO	BP	0.75	10.5	0.1	13.5	1
2BPPR07	F	NO	NO	BP	0.75	10.5	0.1	13.5	2
2BTPR01	F	NO	NO	BT	0.75	10.5	0.1	13.5	2
2BTPR02	F	NO	NO	BT	0.75	10.5	0.1	13.5	5
2BTPR03	F	NO	NO	BT	0.75	10.5	0.1	13.5	5

BOT=Botanical Gardens Halle
PR=Thüringer Bahn
KR=Kröllwitz
BAD=Bad Lauchstädt
ELE=Teutschenthal
FR=Friedeburg
SS=Salziger See
KL=Klobikau
AM=Amsdorf

BP=Bombus pascuorum
BT=Bombus terrestris
BL=Bombus lapidarius

F=Female
M=male

2BTPR04	F	NO	NO	BT	0.75	10.5	0.1	13.5	3
2BTPR05	F	NO	NO	BT	0.75	10.5	0.1	13.5	3
2BTPR06	F	NO	NO	BT	0.75	10.5	0.1	13.5	4
2BTPR07	F	NO	NO	BT	0.75	10.5	0.1	13.5	3
2BLKRO1	F	NO	NO	BL	0.23	13	0.09	12.45	3
2BLKRO2	F	YES	YES	BL	0.23	13	0.09	12.45	3
2BLKRO3	F	YES	NO	BL	0.23	13	0.09	12.45	1
2BLKRO4	F	NO	NO	BL	0.23	13	0.09	12.45	5
2BLKRO5	F	YES	NO	BL	0.23	13	0.09	12.45	5
2BLKRO6	F	NO	YES	BL	0.23	13	0.09	12.45	1
2BLKRO7	F	NO	NO	BL	0.23	13	0.09	12.45	1
2BLKRO8	F	YES	NO	BL	0.23	13	0.09	12.45	2
2BPKRO1	F	NO	NO	BP	0.23	13	0.09	12.45	5
2BPKRO2	F	NO	NO	BP	0.23	13	0.09	12.45	3
2BPKRO3	F	NO	NO	BP	0.23	13	0.09	12.45	1
2BPKRO4	F	NO	NO	BP	0.23	13	0.09	12.45	1
2BPKRO5	F	NO	NO	BP	0.23	13	0.09	12.45	5
2BPKRO6	F	NO	NO	BP	0.23	13	0.09	12.45	3
2BPKRO7	F	NO	NO	BP	0.23	13	0.09	12.45	1
2BPKRO8	F	NO	NO	BP	0.23	13	0.09	12.45	3
2BPKRO9	F	NO	NO	BP	0.23	13	0.09	12.45	3
2BPKRO10	F	NO	NO	BP	0.23	13	0.09	12.45	4
2BPKRO11	F	NO	NO	BP	0.23	13	0.09	12.45	5
2BPBAD1	F	NO	NO	BP	-0.12	7.5	0.08	20.15	5
2BPBAD2	F	YES	NO	BP	-0.12	7.5	0.08	20.15	5
2BPBAD3	F	NO	NO	BP	-0.12	7.5	0.08	20.15	4
2BPBAD4	F	NO	NO	BP	-0.12	7.5	0.08	20.15	4
2BPBAD5	F	NO	NO	BP	-0.12	7.5	0.08	20.15	1
2BPBAD6	F	NO	NO	BP	-0.12	7.5	0.08	20.15	2
2BPBAD7	F	NO	NO	BP	-0.12	7.5	0.08	20.15	2
2BPBAD8	F	NO	NO	BP	-0.12	7.5	0.08	20.15	3
2BPBAD9	F	NO	NO	BP	-0.12	7.5	0.08	20.15	2
2BPBAD10	F	NO	NO	BP	-0.12	7.5	0.08	20.15	3
2BPBAD11	F	NO	NO	BP	-0.12	7.5	0.08	20.15	1
2BPBAD12	F	NO	NO	BP	-0.12	7.5	0.08	20.15	2
2BPBAD13	F	NO	NO	BP	-0.12	7.5	0.08	20.15	5
2BPBAD14	F	NO	NO	BP	-0.12	7.5	0.08	20.15	1
2BPBAD15	F	NO	NO	BP	-0.12	7.5	0.08	20.15	3
2BPBAD16	F	NO	NO	BP	-0.12	7.5	0.08	20.15	2
2BLBAD1	F	NO	NO	BL	-0.12	7.5	0.08	20.15	1
2BLBAD2	F	YES	NO	BL	-0.12	7.5	0.08	20.15	3
2BTBAD1	F	NO	NO	BT	-0.12	7.5	0.08	20.15	3
2BTBAD2	F	NO	NO	BT	-0.12	7.5	0.08	20.15	4
2BLELE3	F	NO	NO	BL	-0.90	6.5	0.05	18.45	1
2BLELE4	F	NO	NO	BL	-0.90	6.5	0.05	18.45	3
2BLELE5	F	NO	NO	BL	-0.90	6.5	0.05	18.45	2
2BLELE6	F	NO	NO	BL	-0.90	6.5	0.05	18.45	3
2BLELE7	F	NO	NO	BL	-0.90	6.5	0.05	18.45	3
2BLELE8	F	NO	NO	BL	-0.90	6.5	0.05	18.45	3
2BLELE9	F	NO	NO	BL	-0.90	6.5	0.05	18.45	5
2BLELE10	F	NO	NO	BL	-0.90	6.5	0.05	18.45	3
2BLELE11	F	NO	NO	BL	-0.90	6.5	0.05	18.45	2
2BLELE12	F	NO	NO	BL	-0.90	6.5	0.05	18.45	5
2BTELE1	F	NO	NO	BT	-0.90	6.5	0.05	18.45	5
2BTELE2	F	NO	NO	BT	-0.90	6.5	0.05	18.45	3
2BTELE3	F	NO	NO	BT	-0.90	6.5	0.05	18.45	5
2BTELE4	F	YES	NO	BT	-0.90	6.5	0.05	18.45	5
2BTELE5	F	NO	NO	BT	-0.90	6.5	0.05	18.45	4
2BTELE6	F	NO	NO	BT	-0.90	6.5	0.05	18.45	5
2BLFR1	F	NO	NO	BL	-0.70	11	0.01	3.6	3
2BLFR2	F	NO	NO	BL	-0.70	11	0.01	3.6	3
2BLFR3	F	NO	NO	BL	-0.70	11	0.01	3.6	3
2BLFR4	F	NO	NO	BL	-0.70	11	0.01	3.6	3
2BLFR5	F	NO	NO	BL	-0.70	11	0.01	3.6	4
2BLFR6	F	NO	NO	BL	-0.70	11	0.01	3.6	2
2BLFR7	F	NO	NO	BL	-0.70	11	0.01	3.6	5
2BTFR1	F	NO	NO	BT	-0.70	11	0.01	3.6	2
2BTFR2	F	NO	NO	BT	-0.70	11	0.01	3.6	3
2BTFR3	F	NO	NO	BT	-0.70	11	0.01	3.6	5
2BTFR4	F	NO	NO	BT	-0.70	11	0.01	3.6	1
2BTFR5	F	NO	NO	BT	-0.70	11	0.01	3.6	5
2BTFR6	F	NO	NO	BT	-0.70	11	0.01	3.6	5
2BTFR7	F	NO	NO	BT	-0.70	11	0.01	3.6	4
2BPFR1	F	NO	NO	BP	-0.70	11	0.01	3.6	2
2BPFR2	F	NO	NO	BP	-0.70	11	0.01	3.6	1
2BPFR3	F	NO	NO	BP	-0.70	11	0.01	3.6	4

2BPFR4	F	NO	NO	BP	-0.70	11	0.01	3.6	3
2BPFR5	F	NO	NO	BP	-0.70	11	0.01	3.6	5
2BPFR6	F	NO	NO	BP	-0.70	11	0.01	3.6	3
2BPFR7	F	NO	NO	BP	-0.70	11	0.01	3.6	4
2BLSS1	F	NO	NO	BL	-0.55	10	0.01	6.3	5
2BLSS2	F	NO	NO	BL	-0.55	10	0.01	6.3	5
2BLSS3	F	NO	NO	BL	-0.55	10	0.01	6.3	3
2BLSS4	F	NO	NO	BL	-0.55	10	0.01	6.3	5
2BLSS5	F	NO	NO	BL	-0.55	10	0.01	6.3	5
2BLSS6	F	NO	NO	BL	-0.55	10	0.01	6.3	2
2BLSS7	F	NO	NO	BL	-0.55	10	0.01	6.3	3
2BTSS1	F	NO	NO	BT	-0.55	10	0.01	6.3	5
2BTSS2	F	NO	NO	BT	-0.55	10	0.01	6.3	5
2BTSS3	F	YES	NO	BT	-0.55	10	0.01	6.3	3
2BTSS4	F	YES	NO	BT	-0.55	10	0.01	6.3	5
2BTSS5	F	NO	NO	BT	-0.55	10	0.01	6.3	2
2BTSS6	F	YES	NO	BT	-0.55	10	0.01	6.3	4
2BTSS7	F	YES	NO	BT	-0.55	10	0.01	6.3	4
2BPSS1	F	NO	NO	BP	-0.55	10	0.01	6.3	2
2BPSS2	F	NO	NO	BP	-0.55	10	0.01	6.3	5
2BPSS3	F	NO	NO	BP	-0.55	10	0.01	6.3	2
2BPSS4	F	NO	NO	BP	-0.55	10	0.01	6.3	3
2BPSS5	F	NO	NO	BP	-0.55	10	0.01	6.3	3
2BPSS6	F	NO	NO	BP	-0.55	10	0.01	6.3	3
2BPSS7	F	NO	NO	BP	-0.55	10	0.01	6.3	3
2BPKL1	F	NO	NO	BP	-0.18	12.5	0.09	17.15	5
2BPKL2	F	NO	NO	BP	-0.18	12.5	0.09	17.15	3
2BPKL3	F	NO	NO	BP	-0.18	12.5	0.09	17.15	5
2BPKL4	F	NO	NO	BP	-0.18	12.5	0.09	17.15	4
2BPKL5	F	NO	NO	BP	-0.18	12.5	0.09	17.15	5
2BPKL6	F	NO	NO	BP	-0.18	12.5	0.09	17.15	1
2BPKL7	F	YES	NO	BP	-0.18	12.5	0.09	17.15	3
2BLAM1	F	NO	NO	BL	0.00	16	0.06	8.1	3
2BLAM2	F	NO	NO	BL	0.00	16	0.06	8.1	2
2BLAM3	F	NO	NO	BL	0.00	16	0.06	8.1	5
2BLAM4	F	NO	NO	BL	0.00	16	0.06	8.1	4
2BLAM5	F	NO	NO	BL	0.00	16	0.06	8.1	1
2BLAM6	F	NO	NO	BL	0.00	16	0.06	8.1	3
2BLAM7	F	NO	NO	BL	0.00	16	0.06	8.1	1
2BLAM8	F	NO	NO	BL	0.00	16	0.06	8.1	2
2BLAM9	F	NO	NO	BL	0.00	16	0.06	8.1	1
2BTAM1	F	NO	NO	BT	0.00	16	0.06	8.1	3
2BTAM2	F	NO	NO	BT	0.00	16	0.06	8.1	5
2BTAM3	F	NO	NO	BT	0.00	16	0.06	8.1	5
2BTAM4	F	NO	NO	BT	0.00	16	0.06	8.1	1
2BTAM5	F	NO	NO	BT	0.00	16	0.06	8.1	3
2BLBOT01	M	NO	NO	BL	0.97	20	0.12	24.25	1
2BLBOT02	M	YES	NO	BL	0.97	20	0.12	24.25	2
2BLBOT03	M	NO	NO	BL	0.97	20	0.12	24.25	1
2BLBOT04	M	NO	NO	BL	0.97	20	0.12	24.25	2
2BLBOT05	M	NO	NO	BL	0.97	20	0.12	24.25	2
2BLBOT06	M	NO	NO	BL	0.97	20	0.12	24.25	1
2BLBOT07	M	YES	NO	BL	0.97	20	0.12	24.25	4
2BPBOT01	M	NO	NO	BP	0.97	20	0.12	24.25	1
2BPBOT02	M	NO	NO	BP	0.97	20	0.12	24.25	1
2BPBOT03	M	YES	NO	BP	0.97	20	0.12	24.25	1
2BPBOT04	M	YES	NO	BP	0.97	20	0.12	24.25	5
2BPBOT05	M	NO	NO	BP	0.97	20	0.12	24.25	1
2BPBOT06	M	NO	NO	BP	0.97	20	0.12	24.25	2
2BPBOT07	M	YES	NO	BP	0.97	20	0.12	24.25	3
2BTBOT01	M	YES	NO	BT	0.97	20	0.12	24.25	4
2BTBOT02	M	YES	YES	BT	0.97	20	0.12	24.25	5
2BTBOT03	M	YES	NO	BT	0.97	20	0.12	24.25	3
2BTBOT04	M	NO	NO	BT	0.97	20	0.12	24.25	2
2BTBOT05	M	NO	NO	BT	0.97	20	0.12	24.25	3
2BTBOT06	M	YES	NO	BT	0.97	20	0.12	24.25	2
2BTBOT07	M	NO	NO	BT	0.97	20	0.12	24.25	4
2BLPR01	M	NO	YES	BL	0.75	10.5	0.1	13.5	2
2BLPR02	M	NO	YES	BL	0.75	10.5	0.1	13.5	3
2BLPR03	M	NO	NO	BL	0.75	10.5	0.1	13.5	1
2BLPR04	M	NO	YES	BL	0.75	10.5	0.1	13.5	2
2BLPR05	M	NO	NO	BL	0.75	10.5	0.1	13.5	1
2BLPR06	M	YES	NO	BL	0.75	10.5	0.1	13.5	3
2BLPR07	M	NO	NO	BL	0.75	10.5	0.1	13.5	3
2BLPR08	M	YES	NO	BL	0.75	10.5	0.1	13.5	3
2BTPR01	M	NO	NO	BT	0.75	10.5	0.1	13.5	2

2BTPR02	M	NO	NO	BT	0.75	10.5	0.1	13.5	2
2BTPR03	M	YES	NO	BT	0.75	10.5	0.1	13.5	3
2BTPR04	M	YES	NO	BT	0.75	10.5	0.1	13.5	4
2BTPR05	M	NO	NO	BT	0.75	10.5	0.1	13.5	2
2BTPR06	M	NO	NO	BT	0.75	10.5	0.1	13.5	3
2BTPR07	M	NO	NO	BT	0.75	10.5	0.1	13.5	3
2BPPR01	M	NO	NO	BP	0.75	10.5	0.1	13.5	1
2BPPR02	M	NO	NO	BP	0.75	10.5	0.1	13.5	1
2BLKR01	M	NO	YES	BL	0.23	13	0.09	12.45	1
2BLKR02	M	YES	YES	BL	0.23	13	0.09	12.45	1
2BLKR03	M	YES	NO	BL	0.23	13	0.09	12.45	1
2BLKR04	M	NO	NO	BL	0.23	13	0.09	12.45	2
2BLKR05	M	YES	NO	BL	0.23	13	0.09	12.45	3
2BLKR06	M	NO	NO	BL	0.23	13	0.09	12.45	2
2BLKR07	M	NO	NO	BL	0.23	13	0.09	12.45	2
2BPKR01	M	NO	NO	BP	0.23	13	0.09	12.45	2
2BPKR02	M	NO	NO	BP	0.23	13	0.09	12.45	2
2BLBAD01	M	YES	YES	BL	-0.12	7.5	0.08	20.15	1
2BLBAD02	M	YES	YES	BL	-0.12	7.5	0.08	20.15	4
2BLBAD03	M	NO	NO	BL	-0.12	7.5	0.08	20.15	2
2BLBAD04	M	NO	NO	BL	-0.12	7.5	0.08	20.15	4
2BLBAD05	M	YES	NO	BL	-0.12	7.5	0.08	20.15	1
2BLBAD06	M	NO	NO	BL	-0.12	7.5	0.08	20.15	3
2BLBAD07	M	NO	NO	BL	-0.12	7.5	0.08	20.15	2
2BPBAD01	M	YES	NO	BP	-0.12	7.5	0.08	20.15	5
2BPBAD02	M	YES	NO	BP	-0.12	7.5	0.08	20.15	3
2BPBAD03	M	NO	NO	BP	-0.12	7.5	0.08	20.15	1
2BPBAD04	M	NO	NO	BP	-0.12	7.5	0.08	20.15	1
2BPBAD05	M	NO	NO	BP	-0.12	7.5	0.08	20.15	1
2BPBAD06	M	YES	NO	BP	-0.12	7.5	0.08	20.15	4
2BPBAD07	M	NO	NO	BP	-0.12	7.5	0.08	20.15	3
2BTBAD01	M	YES	NO	BT	-0.12	7.5	0.08	20.15	3
2BTBAD02	M	YES	YES	BT	-0.12	7.5	0.08	20.15	1
2BTBAD03	M	NO	NO	BT	-0.12	7.5	0.08	20.15	1
2BTBAD04	M	NO	NO	BT	-0.12	7.5	0.08	20.15	2
2BTBAD05	M	YES	NO	BT	-0.12	7.5	0.08	20.15	3
2BTBAD06	M	NO	NO	BT	-0.12	7.5	0.08	20.15	4
2BTBAD07	M	NO	NO	BT	-0.12	7.5	0.08	20.15	3
2BLELE01	M	NO	NO	BL	-0.90	6.5	0.05	18.45	2
2BLELE02	M	NO	NO	BL	-0.90	6.5	0.05	18.45	2
2BLELE03	M	NO	NO	BL	-0.90	6.5	0.05	18.45	1
2BLELE04	M	NO	YES	BL	-0.90	6.5	0.05	18.45	1
2BLELE05	M	NO	YES	BL	-0.90	6.5	0.05	18.45	3
2BLELE06	M	NO	NO	BL	-0.90	6.5	0.05	18.45	3
2BLELE07	M	NO	NO	BL	-0.90	6.5	0.05	18.45	2
2BTELE01	M	NO	NO	BT	-0.90	6.5	0.05	18.45	2
2BTELE02	M	YES	NO	BT	-0.90	6.5	0.05	18.45	1
2BTELE03	M	NO	NO	BT	-0.90	6.5	0.05	18.45	3
2BTELE04	M	YES	NO	BT	-0.90	6.5	0.05	18.45	3
2BTELE05	M	YES	NO	BT	-0.90	6.5	0.05	18.45	2
2BTELE06	M	NO	NO	BT	-0.90	6.5	0.05	18.45	3
2BTELE07	M	NO	NO	BT	-0.90	6.5	0.05	18.45	4
2BLFR1	M	NO	YES	BL	-0.70	11	0.01	3.6	1
2BLFR2	M	NO	NO	BL	-0.70	11	0.01	3.6	4
2BLFR3	M	NO	YES	BL	-0.70	11	0.01	3.6	1
2BLFR4	M	NO	NO	BL	-0.70	11	0.01	3.6	3
2BLFR5	M	NO	NO	BL	-0.70	11	0.01	3.6	3
2BLFR6	M	NO	YES	BL	-0.70	11	0.01	3.6	4
2BLFR7	M	NO	NO	BL	-0.70	11	0.01	3.6	2
2BTFR1	M	NO	NO	BT	-0.70	11	0.01	3.6	2
2BTFR2	M	NO	NO	BT	-0.70	11	0.01	3.6	4
2BTFR3	M	NO	NO	BT	-0.70	11	0.01	3.6	1
2BTFR4	M	NO	NO	BT	-0.70	11	0.01	3.6	1
2BTFR5	M	NO	NO	BT	-0.70	11	0.01	3.6	3
2BTFR6	M	NO	NO	BT	-0.70	11	0.01	3.6	1
2BTFR7	M	NO	NO	BT	-0.70	11	0.01	3.6	3
2BPFR1	M	NO	NO	BP	-0.70	11	0.01	3.6	1
2BPFR2	M	NO	NO	BP	-0.70	11	0.01	3.6	3
2BPFR3	M	NO	NO	BP	-0.70	11	0.01	3.6	3
2BPFR4	M	NO	NO	BP	-0.70	11	0.01	3.6	2
2BPFR5	M	NO	NO	BP	-0.70	11	0.01	3.6	1
2BPFR6	M	NO	NO	BP	-0.70	11	0.01	3.6	1
2BLSS1	M	NO	NO	BL	-0.55	10	0.01	6.3	2
2BLSS2	M	NO	NO	BL	-0.55	10	0.01	6.3	3
2BLSS3	M	NO	NO	BL	-0.55	10	0.01	6.3	5
2BLSS4	M	NO	NO	BL	-0.55	10	0.01	6.3	4

2BTSS1	M	YES	NO	BT	-0.55	10	0.01	6.3	4
2BTSS2	M	NO	NO	BT	-0.55	10	0.01	6.3	4
2BTSS3	M	NO	NO	BT	-0.55	10	0.01	6.3	2
2BTSS4	M	NO	NO	BT	-0.55	10	0.01	6.3	4
2BTSS5	M	NO	NO	BT	-0.55	10	0.01	6.3	3
2BTSS6	M	NO	NO	BT	-0.55	10	0.01	6.3	4
2BTSS7	M	NO	NO	BT	-0.55	10	0.01	6.3	3
2BPSS1	M	NO	NO	BP	-0.55	10	0.01	6.3	3
2BPSS2	M	NO	NO	BP	-0.55	10	0.01	6.3	2
2BLKL1	M	NO	NO	BL	-0.18	12.5	0.09	17.15	3
2BLKL2	M	NO	NO	BL	-0.18	12.5	0.09	17.15	3
2BLKL3	M	NO	NO	BL	-0.18	12.5	0.09	17.15	2
2BLKL4	M	NO	NO	BL	-0.18	12.5	0.09	17.15	1
2BLKL5	M	NO	NO	BL	-0.18	12.5	0.09	17.15	4
2BLKL6	M	NO	NO	BL	-0.18	12.5	0.09	17.15	4
2BLKL7	M	YES	NO	BL	-0.18	12.5	0.09	17.15	3
2BTKL1	M	NO	NO	BT	-0.18	12.5	0.09	17.15	4
2BTKL2	M	NO	NO	BT	-0.18	12.5	0.09	17.15	3
2BTKL3	M	NO	NO	BT	-0.18	12.5	0.09	17.15	3
2BTKL4	M	YES	NO	BT	-0.18	12.5	0.09	17.15	4
2BTKL5	M	YES	NO	BT	-0.18	12.5	0.09	17.15	3
2BTKL6	M	NO	NO	BT	-0.18	12.5	0.09	17.15	3
2BTKL7	M	NO	NO	BT	-0.18	12.5	0.09	17.15	1
2BPKL1	M	NO	NO	BP	-0.18	12.5	0.09	17.15	1
2BPKL2	M	NO	NO	BP	-0.18	12.5	0.09	17.15	4
2BPKL3	M	NO	NO	BP	-0.18	12.5	0.09	17.15	2
2BPKL4	M	NO	NO	BP	-0.18	12.5	0.09	17.15	2
2BPKL5	M	NO	NO	BP	-0.18	12.5	0.09	17.15	2
2BPKL6	M	NO	NO	BP	-0.18	12.5	0.09	17.15	1
2BPKL7	M	NO	NO	BP	-0.18	12.5	0.09	17.15	3
2BLAM1	M	NO	NO	BL	0.00	16	0.06	8.1	3
2BLAM2	M	NO	NO	BL	0.00	16	0.06	8.1	4
2BLAM3	M	NO	NO	BL	0.00	16	0.06	8.1	4
2BLAM4	M	YES	YES	BL	0.00	16	0.06	8.1	2
2BLAM5	M	YES	YES	BL	0.00	16	0.06	8.1	2
2BLAM6	M	NO	NO	BL	0.00	16	0.06	8.1	2
2BLAM7	M	YES	NO	BL	0.00	16	0.06	8.1	4
2BTAM1	M	NO	NO	BT	0.00	16	0.06	8.1	3
2BTAM2	M	NO	NO	BT	0.00	16	0.06	8.1	4
2BTAM3	M	NO	NO	BT	0.00	16	0.06	8.1	2
2BTAM4	M	NO	NO	BT	0.00	16	0.06	8.1	1
2BTAM5	M	NO	NO	BT	0.00	16	0.06	8.1	1
2BTAM6	M	NO	NO	BT	0.00	16	0.06	8.1	2
2BTAM7	M	YES	NO	BT	0.00	16	0.06	8.1	3
